# Recent advances in the delivery of natural-product antioxidants for the treatment of breast cancer

**DOI:** 10.3389/fbioe.2026.1764759

**Published:** 2026-05-08

**Authors:** Fatema Eissa Almansoori, Sungmun Lee

**Affiliations:** 1 Department of Biomedical Engineering and Biotechnology, Khalifa University of Science and Technology, Abu Dhabi, United Arab Emirates; 2 Healthcare Engineering Innovation Group, Khalifa University of Science and Technology, Abu Dhabi, United Arab Emirates; 3 Khalifa University Center for Biotechnology, Khalifa University of Science and Technology, Abu Dhabi, United Arab Emirates

**Keywords:** antioxidants, antioxidant therapy, breast cancer, drug delivery systems, natural products, reactive oxygen species, targeted delivery

## Abstract

Breast cancer is one of the main causes of cancer-related death worldwide in women. Conventional treatment of breast cancer, including surgery and chemotherapy, face challenges such as tumor resistance, damaged organs due to the cytotoxicity of anticancer drugs, and poor drug circulation. Recently, several drug delivery vehicles have been developed for targeted breast cancer therapy enhancing drug bioavailability, solubility, and penetration while minimizing side effects and toxicity. In addition to chemotherapeutic drugs, antioxidants derived from plants have gained attention as promising agents for the treatment of breast cancer. This review explores the effect of natural product antioxidants on breast cancer and summarizes state-of-the-art studies utilizing various drug delivery systems to enhance the delivery of antioxidants to breast cancer.

## 1 Introduction

Cancer is the leading cause of death worldwide. Around 2.0 million people are expected to be diagnosed with cancer in the US in 2025 (Siegel et al., 2025). In females, breast cancer (BC) is one of the most common cancer types with over two million cases recorded in 2022 according to the World Health Organization (WHO) (Zhang et al., 2025b). In addition, it is the main reason for cancer-related deaths in women. Risk factors of breast cancer are classified into modifiable and non-modifiable factors such as sex, age, family history, race, and genetic mutations. Breast cancer occurs mostly in women with age above 50 and in all ages in case of a family history. Mutations in BRAC1 and BRAC2 genes contribute significantly to increasing the risk of breast cancer. Moreover, TP53, CDH1, PTEN, and STK11 are highly penetrant genes associated with breast cancer. Modifiable factors include chosen drugs, physical activity, diet, and exposure to chemicals. Healthier diets, less exposure to toxic chemicals, and regular physical activity are known to reduce the risk of getting cancer. In addition, certain drugs were shown to increase the risk of breast cancer such as diethylstilbestrol during pregnancy (Łukasiewicz et al., 2021).

Modern molecular pathology classifies breast cancer into 5 subtypes based on gene expression profiling: normal-like BC, luminal A and B, triple-negative breast cancer (TNBC), and human epidermal growth factor receptor 2 (HER2) enriched (Pal et al., 2024). As an alternative, breast cancer can be divided into four major groups according to the immunohistochemistry expression of hormone receptors: progesterone receptor positive (PR+), estrogen receptor positive (ER+), TNBC, and human epidermal growth factor receptor positive (HER2+) (Orrantia-Borunda et al., 2022). Both luminal A and B are ER+; however, proliferation rate or Ki-67 expression is different. Luminal A has a lower Ki-67 expression level, which makes them less aggressive and more responsive to hormone therapy. TNBC is an aggressive type of breast cancer that does not have receptors for estrogen (ER-), progesterone (PR-), or HER2(HER2-). Therefore, TNBC is not responsive to hormones and HER2-targeted therapies. These subtypes can significantly impact patients’ response to treatment due to their molecular differences which lead to intra-tumor heterogeneity. Surgery, radiotherapy, and chemotherapy are common treatment strategies for breast cancer. Other treatment options include immunotherapy, hormonal therapy, and gene therapy. Even though the surgery technique was effective, several complications arose. Similarly, chemotherapy and radiation are often associated with serious side effects such as healthy cells damage due to non-specificity and high toxicity (Wasserman et al., 2022).

Cancer cells often exhibit oxidative stress due to continuous production of reactive oxygen species (ROS), resulting in DNA, lipids, and protein damage. Through high levels of ROS in its microenvironment, cancer can alter signaling pathways and survive immune attacks. Normally oxidative stress leads to cell death, but cancer cells survive using antioxidant systems to maintain redox balance. Antioxidants are molecules that can decrease the amount of intracellular ROS. Enzymes (e.g., catalase, superoxide dismutase, and glutathione peroxidases) and small molecules (e.g., NADPH and glutathione) are endogenous antioxidants. Exogenous antioxidants include dietary compounds such as minerals and vitamins A, C and E, and pharmacological agents such as cysteine donor N-acetylcysteine (NAC). The damaging potential of ROS initiated the idea of antioxidant therapy leading to increased use of antioxidants among high-risk individuals and cancer patients (Schmidt et al., 2025; Singh and Manna, 2022). However, antioxidants’ clinical efficacy is limited due to their low bioavailability at the cancer site, low solubility, and rapid degradation (Ahmad et al., 2024).

Over the past decade, researchers have developed various novel techniques to enhance breast cancer treatment and avoid side effects associated with conventional treatment. Drug delivery systems such as liposomes, polymeric hydrogels, and inorganic carriers have been designed and implemented to deliver anti-cancer and antioxidant agents with higher specificity and less cytotoxicity. Targeted drug administration can reduce off-target effects and treat metastatic tumors, which will increase the survival rate of cancer patients (Liu et al., 2021; Senapati et al., 2018; Sezgin-Bayindir et al., 2023). Moreover, various drug delivery systems can be used to deliver antioxidants to the cancer microenvironment addressing limitations associated with antioxidant therapy by enhancing their bioavailability and solubility (Sezgin-Bayindir et al., 2023). This review explores antioxidants roles in breast cancer focusing on plant-based antioxidants and discusses how different drug delivery systems enhance antioxidant-based breast cancer treatment.

## 2 Antioxidants mechanism in breast cancer

### 2.1 Reactive oxygen species in microenvironment of breast cancer

Reactive oxygen species is a term used to describe a family of reactive oxidants that consists of oxygen (Sies et al., 2022). The three primary species of ROS, produced by the reduction of molecular oxygen, are hydrogen peroxide ( ${\text{H}}_{2}{\text{O}}_{2}$ ), superoxide anions ( ${\text{O}}_{2}^{\cdot }\text{– }$ ), and hydroxyl radical ( ${\text{HO}}^{\cdot }$ ). Hypochlorous acid ( HOCl ), nitric oxide ( NO ), and peroxynitrite ( ${\text{ONOO}}^{-}$ ) are oxidizing molecules that make them part of the ROS family. A family of nitrogen-containing molecules is called reactive nitrogen species (RNS). RNS also includes radicals, which makes them reactive, and examples of RNS are nitric oxide radical (^⋅^NO), peroxynitrite ( ${\text{ONOO}}^{-}$ ), and nitrogen dioxide radical (^⋅^NO_2_) (De Almeida et al., 2022).

**FIGURE 1:**
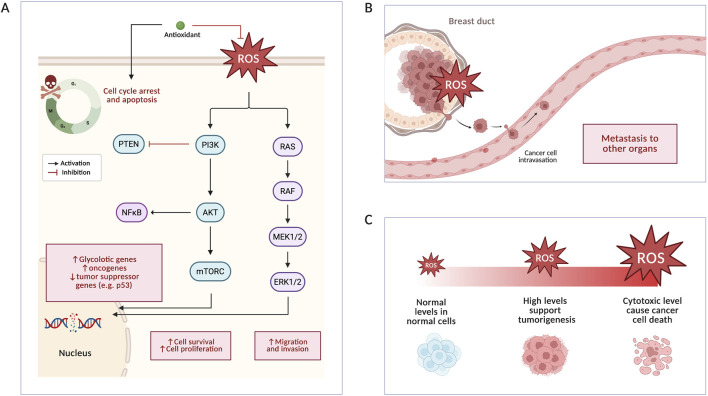
Effect of ROS on breast cancer. **(A)** Pathways that ROS suppress and activate, affecting breast cancer proliferation and the effect of antioxidants. **(B)** ROS production leads to migration of cancer cells from the breast duct to other organs. **(C)** Different levels of ROS have different effects on cells.

Both ROS and RNS play an important role in cellular signaling, inflammation, and defense mechanisms. ROS also contribute to the natural ageing process by inducing apoptosis and cell differentiation. Oxidative stress and high levels of free radicals in cells can cause cell damage (Jakubczyk et al., 2020). While increased metabolic activity, acidosis, and hypoxia trigger ROS production, cancer cells maintain ROS at a level that allows its survival avoiding toxicity and apoptosis. In breast cancer, ROS induce stem cell differentiation and epithelial mesenchymal transition (EMT), promoting breast cancer cells survival (Figure 1). Additionally, ROS drive preprogramming of tumor cells by increasing glycolytic genes expression, transforming oxidative metabolism to glycolytic metabolism. Through hypermethylation, the production of ROS induces mutations assisting in BC development by silencing tumor suppressor genes and activating oncogenes. In addition to metabolic and genetic changes, ROS production alters major pathways in breast cancer. Cell survival and proliferation are promoted by phosphoinositide 3-kinase (PI3K)/protein kinase B (AKT) activation and phosphatidylinositol 3,4,5-trisphosphate 3-phosphatase (PTEN) inhibition through ROS production. Moreover, rat sarcoma (RAS) and mitogen-activated protein kinase (MAPK) pathways are activated which assist in metastasis (Malla et al., 2021). Figure 1A shows the effect of ROS on major BC signal processes. Figure 1B shows the association between ROS and metastasis.

Interestingly, a tumor’s progression or suppression is determined by ROS levels in the tumor microenvironment (TME) (Figure 1C) (Zhong and Tang, 2024). For instance, new blood vessels grow as a result of activating hypoxia inducible factor alpha (HIF- α ) when ROS level is low. Medium level of ROS causes cell cycle dysregulation and proinflammatory cytokine production as a result of activating the MAPK pathway. While cancer promoting mutations accumulate and enhances proliferation at a higher level of ROS, excess intolerable ROS leads to oxidative damage disrupting the DNA, proteins, and lipids (Gupta et al., 2022). Therefore, researchers targeted ROS as a therapy strategy by either inducing stress in the TME or balancing ROS levels. In fact, ROS regulation is essential to avoid oxidative stress and inhibit signaling pathways that allow cancer proliferation and transition (Perillo et al., 2020).

Various antioxidant systems in cells work to control ROS such as enzymes which include catalase and glutathione peroxidases, as well as superoxide dismutase (SODs) (Cheung and Vousden, 2022). Antioxidants are molecules that can donate a proton and an electron which scavenge reactive oxygen and free radicals protecting cells against damage (Gulcin, 2025). It is essential to note that antioxidant systems are also used in cancer cells to maintain ROS levels that support tumor survival and proliferation as a response to oxidative stress. Oxidative stress occurs when the equilibrium between antioxidants and prooxidants is disrupted as a result of reduction in antioxidants or increase in ROS levels (Gulcin, 2025; Barnieh et al., 2025). Whereas antioxidant systems’ role in cancer seems controversial, several studies showed the effectiveness of both endogenous and exogenous antioxidants in suppressing cancer, improving chemotherapy outcomes, and overall patients’ survival.

### 2.2 The effect of antioxidants from natural products on breast cancer

Over the past few years, several antioxidants derived from natural products have been used in cancer therapy and have attracted attention for their anticancer, anti-inflammatory, and antibacterial effects, with minimal side effects. In addition to curcumin, which has been widely used in cancer research, other types of polyphenols (e.g., resveratrol and flavonoids such as quercetin and epigallocatechin gallate (EGCG)) and carotenoids (e.g., beta-carotene) are receiving growing recognition in research. Enormous studies have shown that curcumin, a polyphenol extracted from turmeric plant, possesses great anticancer, anti-inflammatory and antioxidant properties. In addition to treating breast cancer, turmeric has been widely used to treat liver disease, inflammatory disorders, and various cancer types due to its safety and efficacy (Zoi et al., 2021). In breast cancer, curcumin was found to inhibit the expression of MAPK, Akt, and HER2, suppressing tumor proliferation (Hu et al., 2018; Chen et al., 2014). Chen et al. (2014) demonstrated a novel mechanism in which curcumin disrupts major pathways for DNA repair in ER + breast cancer cells (MCF-7 cells). The study showed that flap endonuclease 1 (Fen1), a DNA nuclease essential for BC development, can be downregulated by curcumin thereby inhibiting tumor growth (Chen et al., 2014). Several studies have shown that miRNAs are considered a potential target for breast cancer therapy. By suppressing the p300/miR-142-3p/PSMB5 axis, curcumin inhibits proteosome activity in triple-negative breast cancer, which significantly slows down cell proliferation (Liu et al., 2020). Additionally, miRNAs are another potential therapeutic agent for cancer. It showed its efficacy by suppressing p300/miR-142-3p/PSMB5 axis that at same time, curcumin target by inhibiting the proteosomes activity in triple-negative BC which significantly slows down cell proliferation (Liu et al., 2020). Moreover, curcumin inhibits TNBC cells’ migration and proliferation by altering miR-648-5p expression, which is upregulated in TNBC cells, and its target genes (Kaya et al., 2024). In 4T1 mouse models, curcumin inhibited tumor growth, improved muscle function, and alleviated mitochondrial dysfunction through the activation of NF- κ B pathway (Zhang et al., 2022). Interestingly, curcumin, when combined with resveratrol, was shown to induce apoptosis by disrupting key signaling pathways, including NF- κ B, MAPK, and Akt. Alsikhan demonstrated the synergistic effect of curcumin and resveratrol, tested on MCF-7 and MDA-MB-231 cells, through modulation of long non-coding RNAs (Alsaikhan, 2025).

Resveratrol (RES) is a non-flavonoid polyphenol found in berries, grapes, pines, and peanuts reported to be anti-inflammatory, antioxidant, and anti-cancer (Ko et al., 2017). Studies demonstrated that RES suppresses the proliferation of ER+ and triple-negative BC by inhibiting ERK1/2 pathways (Zhang et al., 2025a; Hu et al., 2019). At the genetic level, RES was found to regulate gene expression of BRCA1, inhibiting BC migration and proliferation and inducing apoptosis (Sahu, 2024). Resveratrol not only induces apoptosis in BC cells but also reduces cisplatin, a cancer drug, side effects and improves its efficacy in MDA231 cells. Moreover, it has been demonstrated that the synergy of resveratrol and cisplatin regulates EMT and inhibits cell migration through ERK, Akt, and NF- κ B (Yang et al., 2021b). In addition to resveratrol, green tea catechins such as epicatechin (EC), and epigallocatechin-3-gallate (EGCG) have shown promise in cancer research (Cheng et al., 2020). Catechins in green tea (a flavonoid polyphenol) possess scavenging ROS antioxidant properties as a result of large numbers of hydroxyl groups present, decreasing free radicals in cells and tissues (Farhan, 2022). Santos et al. (2021) found that green tea extract reduces mutant p53 expression, a tumor suppressor mutated in over 50% of cancer types, in MDA-MB-231 cells. Interestingly, the study showed that in MCF-7, green tea extract enhances p53 expression, inducing cytotoxicity and inhibiting migration (Santos et al., 2021). The ability of antioxidants, particularly green tea catechins, to suppress tumors through different mechanisms based on p53 status, proves their efficacy as a personalized medicine, which is a great advantage in cancer research. Epicatechin was tested separately by Pérez-Durán et al. (2023) and was shown to increase anti-metastatic gene expression, reduce cell migration, and decrease tumor cell survival through Akt/mTOR inhibition and AMPK activation. When interacted with common chemotherapeutics such as Taxol and cisplatin, tea catechins resulted in a significant reduction in the viability of TNBC cells while showing no effect on non-cancerous cells. Moreover, catechins, when compared to chemotherapeutics, induced more apoptosis with minimal toxicity to healthy tissues (Raad et al., 2024). Overall studies showed that natural antioxidants counteract oxidative stress by neutralizing free radicals and ROS through the inhibition or activation of specific signaling pathways and genes that are involved in breast cancer migration and proliferation (Table 1).

**TABLE 1:** Effect of common plant-derived antioxidants on breast cancer.

Antioxidant	Evaluation model	Mechanism	Main effect	Reference
Curcumin	MCF-7	Fen1 downregulation	DNA repair pathways disruption	Chen et al. (2014)
Curcumin	MDA-MB-231	p300/miR-142-3p/PSMB5 axis	Inhibit proteosomes activity	Liu et al. (2020)
Curcumin	MDA-MB-231	miR-638-5p modulation	Inhibit cell proliferation	Kaya et al. (2024)
Curcumin	4T1 mouse model	NF- κ B pathway activation	Inhibition of tumor growth	Zhang et al. (2022)
Curcumin, Resveratrol	MCF-7, MDA-MB-231	Autophagy activation	Enhanced ferroptosis	Alsaikhan (2025)
Resveratrol	MDA-MB-231	ERK1/2 regulation	Inhibit cell proliferation	Zhang et al. (2025a)
Resveratrol	MCF-7, T47D	ERK1/2 regulation	Inhibit cell proliferation	Hu et al. (2019)
Resveratrol	MCF-7, MDA-MB-231, MCF-10A, T47D	DNA methylation, MBD2-BRCA1 axis	Regulation of BRCA1	Sahu (2024)
Resveratrol	MDA-MB-231 xenograft	Akt, ERK, EMT	Enhances cisplatin effect	Yang et al. (2021b)
Green tea extract	MCF-7, MDA-MB-231	P53 regulation	Increased p53 expression and decreased mutant p53 expression	Santos et al. (2021)
Epicatechin	4T1 cells	Akt/mTOR inhibition	Inhibit cell migration	Pérez-Durán et al. (2023)
Green tea, rosemary	MDA-MB-231	Mitochondrial depolarization	Apoptosis	Raad et al. (2024)

Although antioxidants showed efficacy *in vitro*, delivery through complex biological systems can be challenging and has several limitations. Treatment efficacy is limited by antioxidants’ poor solubility, low bioavailability, rapid release, and poor permeability (Yang et al., 2020). Moreover, in oral delivery, antioxidants may lose much of their functionality due to digestive enzymes and the extremely acidic pH of the complex gastrointestinal tract (GIT), making delivery to the tumor site challenging (Lou et al., 2023). At the tumor site, the heterogeneity of the breast cancer microenvironment, consisting of different types of cells, may affect the efficacy of antioxidants. Moreover, hypoxia, high levels of ROS, and acidosis in the TME of breast cancer can affect antioxidant stability. To overcome these limitations, antioxidants are loaded within a drug delivery system to prevent their degradation and enhance their bioavailability (Nisar and Wan, 2025). The ability to engineer and optimize drug delivery systems with unique characteristics allows for prolonged and controlled delivery of the antioxidants, which reduce off-target effects and improve treatment outcomes (Yu et al., 2022).

## 3 Drug delivery systems for antioxidant delivery to breast cancer

**FIGURE 2:**
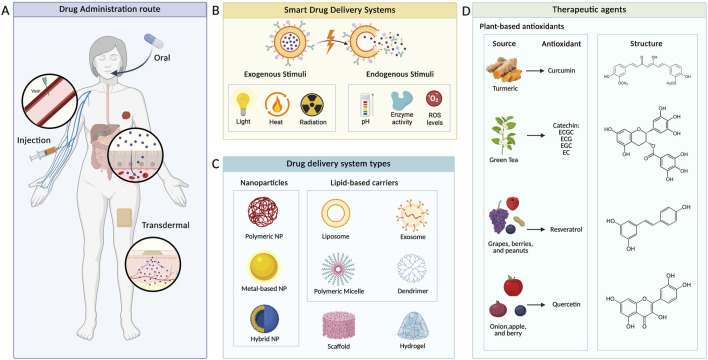
Classification of drug delivery systems. **(A)** Most common drug administration routes to deliver drugs to the target site. **(B)** Examples of exogenous and endogenous stimuli that can trigger drug release. **(C)** Different drug delivery systems as classified in this review. **(D)** Source and structure of the most common plant-based antioxidants used in cancer therapy.

Over the past few decades, drug delivery systems have been developed to address the limitations of antioxidants from natural products, including low bioavailability, poor solubility, and rapid release. To deliver antioxidants efficiently to the site of breast cancer, researchers developed various drug delivery systems, including hydrogels, nanoparticles, micelles, dendrimers, and liposomes. Drug delivery systems can be classified based on administration routes (e.g., oral, transdermal, and intravenous) (Figure 2A), size, material used (e.g., natural and synthetic), crosslinking (e.g., physical and chemical), stimuli (pH, light, and temperature) (Figure 2B), and type of carrier (Figure 2C) (Tang et al., 2023; Pei et al., 2024). In this section, drug carriers used to enhance antioxidant delivery (e.g., curcumin, illustrated in Figure 2D) are classified by type and material.

### 3.1 Nanoparticles

**FIGURE 3:**
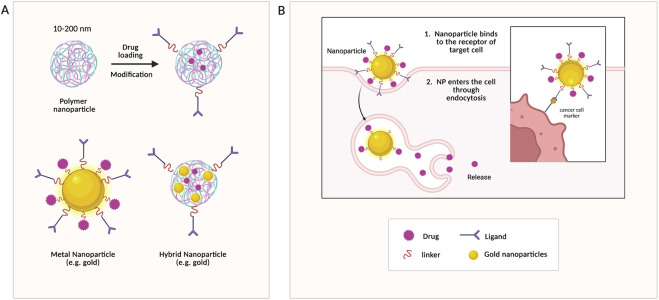
Nanoparticles: structure, modification, and mechanism of action. **(A)** Structure of different classes of nanoparticles. **(B)** Targeted delivery mechanism of gold nanoparticles.

Nanoparticles (NPs) are the most common drug carriers for the delivery of plant-based antioxidants in breast cancer. Several types of nanoparticles have been utilized in cancer research (Figure 3), including polymeric, metal-based (e.g., silver, iron oxide), and hybrid nanoparticles (Figure 3A), due to their small size (10–200 nm), which allow them to penetrate tumors (Altammar, 2023).

### 3.2 Polymeric NPs

Polymeric nanoparticles were shown to improve the sustained intracellular retention and cellular uptake of antioxidants, enhancing treatment efficacy (Araújo et al., 2023). Moreover, it has been demonstrated that polymer-based nanoparticles enhance drug accumulation at the tumor site through improved permeation and retention (EPR). The most common polymers used in recent research are chitosan (CS), hyaluronic acid (HA), poly(ethylene glycol) (PEG), polylactic acid (PLA), and poly(D, L-lactic-co-glycolic acid) (PLGA) (Li et al., 2017).

Chitosan (CS) is a natural polysaccharide derived from chitin, which can be found in algae, insects, fungi, and mollusks (Cheung et al., 2015; Thakur et al., 2023). Bozorgi et al. (2023) were the first to synthesize resveratrol-encapsulated chitosan nanoparticles for triple-negative breast cancer. The half-maximal inhibitory concentration (IC50) values were significantly lower for chitosan nanoparticles loaded with RES than for free RES, indicating enhanced RES bioavailability, solubility, and controlled release due to nanoparticle encapsulation. Resveratrol, as a potent antioxidant and in combination with CS, resulted in increased p53 expression and demonstrated a significant apoptotic effect against TNBC cells (Bozorgi et al., 2023). Similarly, Abdel-Hakeem et al. (2021) used chitosan to load curcumin in chitosan/protamine nanoparticles for BC therapy. The study showed that in MCF-7 cells, curcumin-loaded NPs reduced proinflammatory cytokines, including TNF- α , NF- κ B, and IL-6, and downregulated Bcl2 expression, thereby inhibiting tumor growth (Abdel-Hakeem et al., 2021). Nematollahi et al. (2021) presented a pH-responsive novel nanocomposite based on chitosan, polyvinylpyrrolidone (PVP), and γ -alumina to encapsulate quercetin, a flavonoid with anti-inflammatory and antioxidant properties found in vegetables and fruits (Aghababaei and Hadidi, 2023). Adding γ -alumina nanoparticles resulted in enhanced entrapment efficiency (from 88% to 95.5%), swelling ratio, and pH responsiveness. *in vitro* studies demonstrated that the developed nanocomposite successfully induced apoptosis in MCF-7 cells with reduced side effects (Nematollahi et al., 2021). Another study developed nanoparticles that are not only pH responsive but also respond to environmental ROS levels. However, the study used a polymer known as PCPP, or polyethylene glycol–glycol-2,5-dihydro-4-methyl-2,5-dioxo-3-furanpropanoic-polyethyleneimine-ox-polycaprolactone (PEG-CDM-PEI-ox-PCL), for the synthesis of curcumin-loaded nanoparticles. Curcumin, as a hydrophobic agent, can be loaded into the core of PCPP, which self-assembles into nanoparticles. Intracellular ROS levels trigger the release of curcumin and breakdown of “ox-PCL”. Moreover, the acidic BC microenvironment leads to detachment of polyethylene glycol (PEG), thereby enhancing the drug carrier’s specificity. In addition to antitumor effects through cell cycle arrest demonstrated *in vitro*, *in vivo* studies showed increased curcumin concentration at the tumor site, validating the targeting capability of curcumin dual-responsive nanoparticles and its antioxidant effect (Li et al., 2022a).

In addition to chitosan, PLGA is used to synthesize nanoparticles for the delivery of antioxidants from natural products to breast cancer. Chatterjee et al. (2024) formulated PLGA-based nanoparticles for the treatment of TNBC loading Carnosic acid (CA), a phenolic antioxidant present in high levels in rosemary and established as a chemotherapeutic agent (Loussouarn et al., 2017). CA alone induces oxidative stress in cancer cells by inhibiting the activity of endogenous antioxidants, including catalase and glutathione. Compared with free CA, CA-PLGA nanoparticles induced greater apoptosis, driven by enhanced CA concentration at the tumor site and improved uptake (Chatterjee et al., 2024). The ability of antioxidants to scavenge free radicals in healthy tissues while inducing oxidative stress in cancer cells suggests their potential as therapeutic agents for cancer. Building on the findings of Chatterjee et al. (2024), Dutta et al. (2022) developed Asiatic acid-loaded PLGA-based nanoparticles for breast cancer therapy. Asiatic acid (AA) is a triterpenoid antioxidant found in *Centella asiatica* (L.) Urb, and was shown to induce apoptosis by p53 upregulation, inhibition of PI3K/Akt/mTOR, and downregulation of ERK1 (Chen et al., 2025). The AA-PLGA nanoparticles exhibited sustained release for up to 9 days while maintaining stability. Like Chatterjee et al. (2024), Dutta et al. (2022) emphasized the dual role of antioxidants in which AA-loaded NPs induced apoptosis in cancer cells (MCF-7) by increasing ROS levels, causing oxidative stress and mitochondrial dysfunction. Moreover, no cytotoxicity was observed in normal cells, demonstrating the safety of AA-PLGA nanoparticles for breast cancer treatment (Dutta et al., 2022).

Hyaluronic acid (HA) is a mucopolysaccharide that occurs naturally in the human body, specifically in mucus and joint fluid known for its high viscoelasticity, biodegradability, and good biocompatibility (Buckley et al., 2022; Huang and Huang, 2018).Bhattacharya et al. (2020) developed thymoquinone-loaded nanoparticles based on HA as well as F127 and P123 Pluronic copolymers.*in vitro* results showed TNBC cell migration inhibition via microRNA-361 inhibition, VEGF-A deregulation, and downregulation of Rac1 and RhoA. Therefore, HA-based nanoparticles loaded with thymoquinone showed notable anti-angiogenesis and anti-metastasis effects both *in vitro* and *in vivo* (Bhattacharya et al., 2020). Kumari and his team developed PGMD(poly-glycerol-malic acid-dodecanedioic acid)/curcumin nanoparticles for breast cancer therapy. The nanoparticles were formulated at different ratios of PGMD and curcumin, yielding an encapsulation efficiency range of 75%–81%. MTT (3-[4,5-dimethylthiazol-2-yl]-2,5 diphenyl tetrazolium bromide) assay results showed that curcumin-loaded NPs induced apoptosis in both MDA-MB-231 and MCF-7 cells. As an antioxidant, curcumin induced apoptosis via caspase-9 overexpression (Kumari et al., 2021).

### 3.3 Metal NPs

Metals such as silver, gold (Figure 3B), platinum, and iron oxide have been used to synthesize drug delivery systems and integrated into biological systems without notable toxicity, attracting research attention (Yaqoob et al., 2020). In Das et al. (2024), the researchers used Curcuma caesia Roxb. (black turmeric) to synthesize gold nanoparticles and evaluated their cytotoxicity in breast cancer cells (MCF-7 and MDA-MB-231). Both black turmeric extract and the AuNPs it mediates demonstrated enhanced toxicity towards TNBC cells, with the AuNPs showing greater toxicity (Das et al., 2024). Jha et al. (2025) highlighted the synthesis of gold nanoparticles using citrus maxima extract, a flavonoid with antimicrobial and anticancer effects. Unlike polymeric nanoparticles in previous studies, metallic NPs were capped with plant extracts, providing stabilization. The findings suggested that AuNPs suppressed migration, as evidenced by downregulation of TGF- β 1, matrix metalloproteinase-9 (MMP-9), and matrix metalloproteinase-2 (MMP-2) which are genes related to metastasis. Moreover, the nanoparticles induced apoptosis in MCF-7 and MDA-MB-231 via cell cycle arrest. Furthermore, the release of gold induced ROS generation, leading to ROS-mediated cytotoxicity and contributing to BC cell apoptosis (Jha et al., 2025). Another study fabricated gold NPs, as well as iron and selenium NPs, using plant extracts, known to possess anti-cancer and antioxidant properties (Shnoudeh et al., 2022). Among the three NPs used in this study, selenium demonstrated cytotoxicity towards MCF-7 cells inducing apoptosis via pathways such as MAPK/Akt and PI3K/Akt/mTOR. While gold and iron NPs showed no cytotoxicity in MCF-7 cells, synthesizing them using plant extracts (green synthesis) enhanced their safety in normal cells, thereby significantly improving overall therapy safety and reducing side effects (Shnoudeh et al., 2022). Mohanta et al. (2022) demonstrated dose-dependent cytotoxicity of seaweed-mediated silver nanoparticles (AgNPs) against TNBC cells, achieving an IC50 value of 344.27 ± 2.56 μ g/mL (Mohanta et al., 2022). Al-Asiri et al. (2024) developed silver nanoparticles from Euphorbia retusa (EU-AgNPs) for breast cancer treatment by a single-step reduction of silver ions. In MCF-7 cells, the nanoparticles induced cytotoxicity with an IC50 value of 40 μ g/mL. Moreover, the EU-AgNPs were shown to increase levels of p53, caspase-3, and caspase-9 (apoptotic marker genes) (Al-Asiri et al., 2024). As demonstrated, metal-based nanoparticles targeted breast cancer by increasing ROS generation acting as pro-oxidant systems, while capping with plant extracts reduce immune rejection and enhance safety and biocompatibility.

### 3.4 Hybrid NPs

Several studies combined polymers and metals to synthesize innovative hybrid nanoparticles that exhibit unique properties addressing various shortcomings of drug delivery systems (Venditti, 2022). Alzahrani et al. (2023) presents a novel nanocomposite made from sodium alginate and iron oxide for the encapsulation of thymoquinone. The results showed that the nanocomposite successfully induced apoptosis in MDA-MB-231 cells through ROS generation, nuclear damage, oxidative stress, PI3K-Akt-mTOR inhibition, and cell cycle arrest (Alzahrani et al., 2023). In another study, the researchers developed nanovehicles comprising chitosan, graphitic carbon nitride, and halloysite for the targeted delivery of quercetin. The inclusion of graphitic carbon nitride (g-C3N4) increased the encapsulation efficiency to 86%. Moreover, the nanocomposites exhibited pH-responsive behavior and stability at neutral pH, owing to the interaction between quercetin and the nanoparticles, resulting in sustained drug release (up to 96 h). Compared to free quercetin, loaded quercetin reduced MCF-7 cell viability and improved apoptosis. Therefore, the study demonstrated excellent biocompatibility, effective loading, and extended quercetin release, thereby significantly enhancing antioxidant therapy via hybrid nanocomposites (Sabzini et al., 2023). Abdouss et al. (2023) highlights a similar trend through green synthesis of a pH-responsive graphitic carbon nitride/polyacrylic acid/chitosan nanocarrier for the delivery of curcumin to breast cancer cells. Similar to Sabzini et al. (2023), the addition of graphitic carbon nitrite enhanced the encapsulation efficiency of curcumin, achieving a controlled release of 96 h. Moreover, graphitic carbon nitride/polyacrylic acid/chitosan nanocarrier eliminated MCF-7 cells more effectively than free curcumin, addressing limitations such as curcumin’s low solubility and short half-life (Abdouss et al., 2023). Given curcumin’s long-standing promise in cancer research, another study encapsulated it in a nanocomposite comprising chitosan, carbon quantum dots, and $Fe_{2}O_{3}$ for breast cancer therapy. The *in vitro* release study showed higher curcumin release at pH 5.4 than at pH 7.4, suggesting a pH-responsive behavior that improves target specificity. Moreover, the study achieved high encapsulation efficiency and controlled drug delivery using the double emulsion method and a chitosan matrix. In MCF-7 cells, the chitosan/carbon quantum dots/ $Fe_{2}O_{3}$ nanocomposite showed a significant reduction in cell viability, suggesting its potential as an alternative for breast cancer therapy (Zoghi et al., 2023). Rajkumar et al. (2025) evaluated the antioxidant activity of Anacardium occidentale extract used to formulate nanocomposites based on chitosan, copper oxide (CuO), and polyvinyl alcohol (PVA). The Cs/PVA/CuO NPs demonstrated antioxidant, antibacterial, and notable anticancer activity, particularly against MCF-7 cells after 24 h of treatment. The synergy of polymers and metals, combined with the use of plant extract, enabled controlled release and high cancer cytotoxicity with minimal side effects, thereby enhancing treatment efficacy and safety (Rajkumar et al., 2025). Table 2 demonstrates the studies mentioned above.

**TABLE 2:** Summary of antioxidant-loaded nanomaterials and their evaluation models.

Material	Antioxidant	Evaluation model	Size (nm)	Encapsulation efficiency (%)	Reference
Chitosan	Resveratrol	MDA-MB-231	—	52.34	Bozorgi et al. (2023)
Chitosan	Curcumin	MCF-7	200	67	Abdel-Hakeem et al. (2021)
PVP/CS/ γ -alumina	Quercetin	MCF-7	141	95.5	Nematollahi et al. (2021)
PCPP	Curcumin	MDA-MB-231, 4T1	—	—	Li et al. (2022a)
PLGA	Carnosic acid	MDA-MB-231	124.9	65.66	Chatterjee et al. (2024)
PLGA	Asiatic acid	MCF-7, MDA-MB-231, BALB/c mice	—	65.63	Dutta et al. (2022)
HA, F127, P123	Thymoquinone	MDA-MB-231, 4T1	19.3	—	Bhattacharya et al. (2020)
PGMD	Curcumin	MCF-7, MDA-MB-231	200	75–81	Kumari et al. (2021)
Gold	Black turmeric	MCF-7, MDA-MB-231	18	—	Das et al. (2024)
Gold	Citrus maxima extract	MCF-7, MDA-MB-231	12–25	—	Jha et al. (2025)
Gold, Iron, Selenium	Punica granatum, Ephedra alata, Pistacia lentiscus extracts	MCF-7	39.16	—	Shnoudeh et al. (2022)
Silver	Gracilaria edulis	MDA-MB-231	62.72	—	Mohanta et al. (2022)
Silver	Euphorbia retusa	MCF-7	10–25	—	Al-Asiri et al. (2024)
Sodium Alginate, iron oxide	Thymoquinone	MDA-MB-231	55	—	Alzahrani et al. (2023)
CS, graphitic carbon nitride, halloysite	Quercetin	MCF-7	454.65	86	Sabzini et al. (2023)
CS, graphitic carbon nitride, polyacrylic acid	Curcumin	MCF-7	315	87	Abdouss et al. (2023)
Chitosan/carbon quantum dots/ ${\text{Fe}}_{2}{\text{O}}_{3}$	Curcumin	MCF-7	227.2	87	Zoghi et al. (2023)
Chitosan, CuO, PVA	Anacardium occidentale extract	MCF-7	48.6–96.2	—	Rajkumar et al. (2025)

### 3.5 Liposomes

**FIGURE 4:**
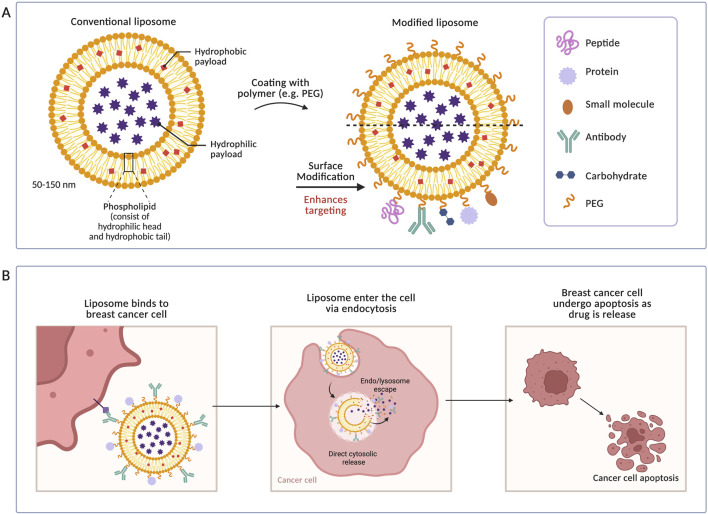
Liposomes for the treatment of breast cancer. **(A)** Structure of conventional and modified liposomes. **(B)** Targeting process of cancer cells using modified liposomes for enhanced targeting and specificity.

Liposomes are widely used in cancer research and have attracted attention as drug delivery vehicles due to their high biocompatibility, ability to load various drugs, and ease of fabrication (Torchilin, 2005; Yang et al., 2021a). Doxorubicin (Dox) and Doxil are liposomal therapies approved and used clinically by the Food and Drug Administration (FDA). Liposomes are created by dispersing synthetic or natural amphipathic lipids in water, resulting in a fat bilayer structure (Mukherjee et al., 2022). Due to its amphipathic nature in solution, hydrophilic and hydrophobic drugs can both be encapsulated in liposomes (Figure 4) (Nsairat et al., 2022). The structure of conventional and modified liposomes is demonstrated in Figure 4A. Figure 4B shows liposomal targeted delivery to breast cancer cells. Therefore, several studies developed liposomes for the co-delivery of antioxidants and chemotherapeutic drugs.

Sharma et al. (2024) developed a chitosan-coated liposomal carrier to orally deliver exemestane (a chemotherapeutic drug) and genistein to breast cancer cells (Sharma et al., 2024). Genistein, an isoflavone found in soybeans, has shown antioxidant and anticancer effects by suppressing Akt and blocking the NF- κ B pathway (Bhat et al., 2021). *in vitro* findings showed that coating with chitosan resulted in high encapsulation effeciency and sustained release protecting the antioxidants from harsh GIT environment. Coated liposomes demonstrated enhanced cytotoxicity and significant antioxidant activity in MCF-7 cells, with no side effects *in vivo*, making them efficient drug carriers for breast cancer (Sharma et al., 2024). Another study co-encapsulated curcumin and docetaxel in liposomes to enhance breast cancer treatment. Compared to free docetaxel and curcumin, loading curcumin and docetaxel within liposomes reduced organ damage through prolonged drug retention time in the body. The developed system demonstrated enhanced anti-tumor effects in mice bearing MCF-7 tumors. Since breast cancer patients commonly exhibit multidrug resistance leading to the failure of chemotherapy treatment, the co-delivery of curcumin and docetaxel was shown to overcome resistance (Ye et al., 2022). Like docetaxel, doxorubicin (DOX) is a chemotherapy drug with cytotoxic properties that depletes cellular antioxidants and severely affects healthy tissues and organs (Radeva and Yoncheva, 2025; Linders et al., 2024). In addition to DNA damage, DOX alters epigenetics and affects mitochondria, leading to diastolic dysfunction primarily due to non-specificity (Li, 2023). Studies have shown that the inclusion of antioxidants such as melatonin, vitamin C, and ferulic acid amplifies DOX cytotoxicity in cancer cells while protecting normal cells (Nayak et al., 2024; Miatmoko et al., 2025).

Ferulic acid is a free radical scavenger with antioxidant, antimicrobial, and anticancer properties (Zduńska et al., 2018; Zheng et al., 2024). In a recent study, researchers loaded ferulic acid and DOX into PEGylated liposomes prepared by the thin-film hydration method. Due to the low bioavailability of ferulic acid and the cytotoxicity of DOX, co-loading them into liposomes is essential for improved therapeutic outcomes. Ferulic acid, as an antioxidant, was shown to improve DOX toxicity in cytotoxicity assays *in vitro*. While PEG chains were shown to enhance liposomes’ colloidal stability by forming a physical barrier, further stability tests are required (Miatmoko et al., 2025). Other than ferulic acid, crocin significantly increased DOX cytotoxicity in MDA-MB-231 cells. Chavoshi et al. (2023) are the first to load crocin, an antioxidant derived from saffron, in liposomes to improve DOX efficacy in TNBC. The liposomes, prepared by thin-layer hydration sonication, showed that crocin enhanced DOX cytotoxicity, demonstrating a similar antioxidant effect to Miatmoko et al. (2025). Moreover, through an apoptotic mechanism, crocin-loaded liposomes showed increased sensitivity of breast cancer cells to DOX.

Apoptosis of triple-negative BC cells by crocin was found to be due to mitochondrial dysfunction, inhibition of metabolic pathways, and protein synthesis regulation (Chavoshi et al., 2023). Xiu et al. (2025) go beyond traditional liposomes through hyaluronic acid (HA) modification and encapsulation within platelet membranes. Research showed that HA assists in the reduction of drug degradation rate and conveys prolonged circulation time *in vivo* (Sharma et al., 2025). Moreover, HA molecules cause endocytosis via the CD44 receptor, promoting liposomes’ internalization into tumor cells (Sun et al., 2024). In this study, the nanocarrier system was formulated using the ethanol injection method: cholesterol, egg yolk lecithin, hyaluronic acid, doxorubicin, and melatonin were dissolved in ethanol to form the initial liposomes, which were subsequently extruded through platelet membranes. Encapsulation of liposomes within platelet membranes was found to enhance specificity and prolong blood circulation. Compared to free drugs and unmodified liposomes, the developed drug carrier inhibited tumor growth and enhanced cell migration suppression. Moreover, inclusion of melatonin improved tumor angiogenesis inhibition by preventing neutrophil extracellular traps (NETs), which are essential for metastasis. In addition to NETs inhibition, combination therapy disrupted the cross-communication between tumor cells, NETs, and cancer-associated fibroblasts (CAFs), inducing tumor cell apoptosis. Therefore, the HA modified liposomes co-loaded with DOX and melatonin can successfully enhance breast cancer therapy, emerging as a promising innovation (Xiu et al., 2025). Overall liposomal combination therapy shows great potential in enhancing breast cancer treatment outcomes. All studies in this section are summarized in Table 3.

**TABLE 3:** Combination of chemotherapy and natural antioxidants via liposomes for breast cancer treatment.

Antioxidant	Drug	Evaluation model	Encapsulation efficiency (%)	Administration route	Reference
Genistein	Exemestane	MCF-7, Wistar rats	77.5	oral	Sharma et al. (2024)
Curcumin	Docetaxel	MCF-7, xenograft mice	98.32	intravenous	Ye et al. (2022)
Ferulic acid	Doxorubicin	MDA-MB 231, T47D, LLC, HeLa	85.99	—	Miatmoko et al. (2025)
Crocin	Doxorubicin	MDA-MB 231	70.2	—	Chavoshi et al. (2023)
Melatonin	Doxorubicin	MCF-7, MRC-5, 4T1	92	intravenous	Xiu et al. (2025)

### 3.6 Hydrogels

**FIGURE 5:**
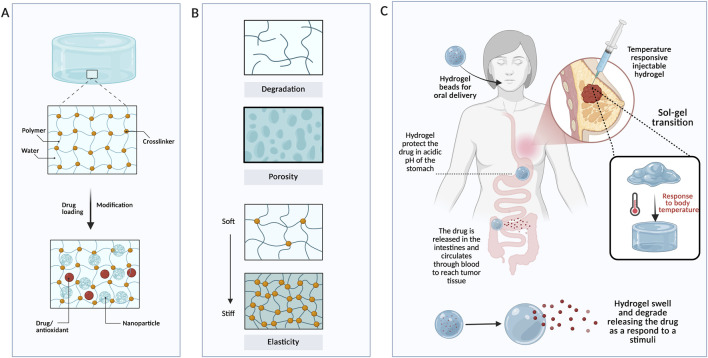
Injectable or oral stimuli-responsive hydrogels for breast cancer treatment. **(A)** Hydrogel structure with and without modification and drug loading. **(B)** Common properties of hydrogels. **(C)** Administration and pathway of a thermo-sensitive injectable hydrogel and a pH-sensitive oral hydrogel as examples of stimuli-responsive hydrogels.

Hydrogels are three-dimensional networks of hydrophilic polymers that can absorb vast amounts of water or dissolved solutes (Figure 5A). In addition to breast cancer, hydrogels are widely used in various biomedical applications due to their unique physical and chemical properties, including flexibility, controllable swelling behavior, porosity, biocompatibility, and biodegradability (Figure 5B) (Kesharwani et al., 2021; Narayanaswamy and Torchilin, 2019). Since hydrogels resemble human tissues—being porous, soft, and high in water content—they have been used in tissue engineering, drug delivery, wound dressing, and 3D cell culture (Ho et al., 2022). In the context of drug delivery, encapsulation of drugs within hydrogels makes them less prone to aggregation and denaturation when exposed to organic solvents. Moreover, hydrophilic drugs can be easily encapsulated in hydrogels due to their high water content, which not only mimics tissue-like environments but also enhances their biocompatibility (Li and Mooney, 2016). The application of a hydrogel specifies its characteristics and design, including the polymer used, size, administration, interactions, and stimuli (Figure 5C). Hydrogels are classified into natural (e.g., hyaluronic acid, collagen, gelatin, chitosan) and synthetic (e.g., polycaprolactone (PCL), PEG, PVA), as well as hybrid, in which a hydrogel is formed using both natural and artificial materials (Rana and De la Hoz Siegler, 2024; Cao et al., 2021). In addition to material-based classification, hydrogels can be classified based on the crosslinking type or interactions forming physical hydrogels (e.g., non-covalent, hydrophilic, and hydrogen bonding), chemical hydrogels, or hybrid crosslinking (a combination of both) (Bashir et al., 2020). The crosslinking ratio is attributed to the release behavior of a hydrogel, resulting in altered swelling kinetics, in which higher crosslinking ratios lead to lower swelling degrees (Yang et al., 2023). Recent hydrogels are becoming smart, sensitive to subtle alterations in their microenvironment and responsive to stimuli, including endogenous factors such as pH, ROS levels, and enzymes, and exogenous stimuli such as temperature, light, and radiation (Neumann et al., 2023; Li et al., 2022b). After designing a hydrogel, it can be delivered via surgical implantation, orally (the most common way in pharmaceuticals), or via local injection (Li and Mooney, 2016). These design mechanisms have been applied across various hydrogel systems to enhance breast cancer therapy and targeted delivery of plant-based antioxidants. Shin et al. (2022) developed an injectable resveratrol-loaded hyaluronic acid hydrogel via click crosslinking to enhance TNBC treatment. Click crosslinking allows the hydrogel to solidify only after injection and intratumor delivery, while remaining in liquid form before injection. Tetrazine (Tet) and trans-cyclooctene (TCO) were used to modify HA and to evaluate their ability to form *in situ* click-crosslinked HA hydrogels loaded with RES (RES-Cx-HA). The findings demonstrated that crosslinking between HA-Tet and HA-TCO yielded Cx-HA with improved biocompatibility. The study addresses the limitations of resveratrol, such as chemical instability, short half-life, and low bioavailability, by injecting the hydrogel and forming a depot in the tumor tissue that could persist for a prolonged period. Specifically, resveratrol was rapidly lost when injected alone or without crosslinking. In contrast, crosslinked hydrogel (Cx-HA) maintained resveratrol in the tumor tissue for an extended period while less than 1% of RES was detected in other organs (e.g., lungs, colon, and kidneys), showing a promising TNBC treatment using antioxidant-loaded HA hydrogels (Shin et al., 2022).

Delivery of RES was further enhanced through a thermosensitive hydrogel developed by Kotta et al. (2022). Poloxamer 407 (Pluronic F-127) is known to form a gel at physiological temperatures and was thus used to make the hydrogel thermosensitive. Eighty percent of resveratrol was released *in vitro* within 6 h, showing enhanced solubility and prolonged release. Moreover, the hydrogel containing nanoemulsified resveratrol demonstrated cytotoxicity in MCF-7 cells, demonstrating its potential as a breast cancer therapy (Kotta et al., 2022). Kundrapu et al. (2025) formulated an injectable BSA pH-responsive hydrogel for targeted delivery of flavonoids (quercetin and taxifolin) to TNBC cells. Bovine serum albumin (BSA) hydrogels are suitable for drug delivery because of their high stability and biocompatibility. In addition to encapsulating quercetin (QUE) and taxifolin (TAX), the study also encapsulates DOX in BSA hydrogel. The entrapment efficiency of QUE, TAX, and DOX was reported to be 93.5, 90, and 91.2% respectively. Moreover, at lower pH 90.8, 95.8, 90.8% of each of the three drugs were released in MDA-MB-468 and MDA-MB-231 triple-negative BC cells. Additionally, all the hydrogels induced ROS-mediated apoptosis in TNBC cells, as evidenced by increased ROS generation and oxidative stress, as well as cell cycle arrest, suggesting that BSA hydrogels are effective drug carriers for antioxidant-based breast cancer therapy (Kundrapu et al., 2025).

## 4 Other drug delivery systems

### 4.1 Micelles

**FIGURE 6:**
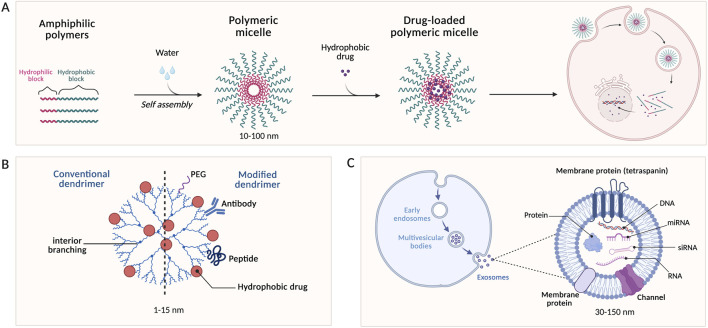
Lipid-based drug delivery systems for breast cancer therapy. **(A)** Amphiphilic polymers self-assemble into polymeric micelles in water and can be loaded with drugs. **(B)** Structure of drug-loaded conventional and modified dendrimers. **(C)** Exosome synthesis, structure, and composition.

Polymeric micelles are drug delivery vehicles that self-assemble from amphiphilic polymers in solution (Figure 6A) (Wang et al., 2023). The properties of the solvent and the morphology of amphiphilic molecules determine micelle formation. Micelles form at a specific concentration of amphiphilic molecules in solution when they are continuously added (Goyal et al., 2017). Polymeric micelles are easier to formulate than other nanocarriers, smaller in size, and have promising solubilization properties (Ghezzi et al., 2021). Since plant-derived antioxidants such as curcumin and resveratrol are hydrophobic and often exhibit poor solubility and low bioavailability, encapsulating them in micelles can address these limitations (Guo et al., 2018). Gregoriou et al. (2021) present an optimized approach to prepare Pluronic F127 polymeric micelles loaded with resveratrol for enhanced breast cancer therapy. The researchers utilized Pluronic F127, which is commonly used in micelle fabrication due to its ability to stabilize hydrophobic drugs and its unique triblock structure consisting of polyethylene oxide (PEO) and polypropylene oxide (PPO). In solution, Pluronic F127 self-assembles into micelles, with hydrophobic PPO forming the inner layer and encapsulating the drug, while PEO forms the outer layer due to its hydrophilic nature. The drug carrier in the study, in addition to Pluronic F127, was made from Vitamin E D- α -Tocopherol poly (ethylene glycol) 1000 succinate (TPGS) to enhance cellular uptake. The results showed that the drug carrier can successfully target BC cells, including MCF-7 and MDA-MB-231, resulting in reduced viability without toxic effects on healthy tissues (Gregoriou et al., 2021). Another study formulated mixed micelles for the co-delivery of curcumin and docetaxel to address drug resistance in BC cells. The micelles were developed from Soluplus and TPGS since they have been shown to improve encapsulation efficiency, drug loading, and tumor targeting. Building on previous combination therapy studies using curcumin, this study further demonstrated curcumin’s ability to enhance docetaxel cytotoxicity against MCF-7/Adr cells, thereby reversing drug resistance. The effectiveness of docetaxel and curcumin-loaded mixed micelles was shown to be attributed to a specific particle size that allowed efficient absorption in the GIT, increased solubility of docetaxel, and curcumin’s role in preventing potential side effects of docetaxel. While the potential of mixed micelles for the treatment of drug-resistant breast cancer has been demonstrated, further research is needed to understand their mechanism of action (Dian et al., 2024).

### 4.2 Dendrimers

In 1978, Fritz Vogtle discovered dendrimers, nanosized, symmetric molecules with monodisperse structures composed of branches (Figure 6B). Since then, researchers have been developing dendrimer drug carriers for enhanced delivery of different drugs (Abbasi et al., 2014). The structure of a dendrimer comprises two layers: an inner layer to improve drug properties (e.g., controlled release, encapsulation efficiency, and toxicity), and an outer layer containing functional groups that are often used for targeting and drug conjugation (Sun et al., 2023). There are two methods to prepare dendrimers: the first is a layer-by-layer approach in which the dendrimer forms from the core outward, known as “divergent” synthesis. “Convergent” synthesis is when the dendrimer grows from the outside to the inside or the core (Smith et al., 2021). In the last 5 years, only one paper has been published on the use of dendrimers for targeted plant-based antioxidant delivery in breast cancer therapy. The study by Matiyani et al. (2023) aimed to develop a dendrimer-based nanocomposite from poly (amido amine) (PAMAM) and graphene oxide (GO) for the delivery of quercetin. PAMAM dendrimers are exceptional dendrimers that have long been used as drug carriers due to their unique properties, such as low cytotoxicity, mono-dispersity, controllable size, and simple fabrication. The study demonstrated that, compared with graphene oxide alone, incorporating PAMAM increased quercetin loading capacity. Additionally, higher amounts of quercetin were released at pH four than at pH 7.4, indicating a pH-responsive system. *in vitro* cytotoxicity tests showed that quercetin-loaded GO-PAMAM dendrimers have successfully accumulated QUE and caused cytotoxicity in MDA-MB-231 cells, showing good distribution and enhanced targeted delivery (Matiyani et al., 2023).

### 4.3 Exosomes

One way cells get rid of waste products is through extracellular vesicles (EVs) called exosomes. Exosomes are small (30–150 nm in diameter) extracellular vesicles derived from membrane-bound compartments within cells known as endosomes (Sharma and Mukhopadhyay, 2024). They assist in cell-cell communication by carrying bioactive cargoes, including DNA, proteins, lipids, mRNA, small interfering RNA (siRNA), microRNA (miRNA), and other signaling molecules (Figure 6C) (Koh et al., 2023). Since exosomes are naturally produced by many cells, they exhibit lower immunogenicity and better biocompatibility than conventional drug delivery systems, such as micelles, dendrimers, liposomes, and polymeric nanoparticles (Koh et al., 2023; Kim et al., 2024). Another advantage that has attracted researchers’ attention is that exosomes can naturally cross the blood-brain barrier (BBB), thereby expanding their potential applications and enhancing the therapeutic efficacy of drugs loaded (Kim et al., 2024). Therefore, exosomes have emerged as a promising drug delivery system, protecting various drugs from metabolism, including plant-derived antioxidants such as curcumin and resveratrol, as demonstrated in González-Sarrías et al. (2022). The study encapsulated both curcumin and resveratrol in exosomes derived from milk, which is a highly recommended source for exosome-based therapies due to its advantages, including inert toxicity, cross-species biocompatibility, low immunogenicity, and superior stability and half-life (Adriano et al., 2021). Curcumin and resveratrol were not detected in mammary tissue when injected freely into female mice, suggesting the need for a drug vehicle to enhance their targeting and biodistribution. In contrast, exosomes successfully delivered both antioxidants to mammary tissues and protected them from metabolism, as demonstrated by measuring the concentrations of curcumin and resveratrol. The anticancer and antiproliferative effects of curcumin and resveratrol were similar when tested on BC cell models. In MCF-7 cells, both resveratrol and curcumin decreased S-phase cells and increased G0/G1 cells, leading to cell cycle arrest. This response was dependent on antioxidant concentration and the cell line used. Moreover, the developed exosomes induced p53-mediated apoptosis in both p53-mutant (MDA-MB-231) and p53-wild (MCF-7) BC cells. Therefore, the study has demonstrated the potential of milk-derived exosomes to enhance the anticancer effects of curcumin and resveratrol, as well as to improve their delivery to breast cancer tissues (González-Sarrías et al., 2022). Another study used plant-based antioxidants to synthesize exosome-like nanovesicles rather than encapsulating them and evaluated their efficacy against metastatic breast cancer. Chen and team isolated for the first time exosome-like nanovesicles from the flowers of the tea plant, labelled TFENs. The exosomes were prepared from lipids, cytosolic proteins, membrane proteins, polyphenols, and flavones via grinding of tea flowers and centrifugation. The effect of TFENs on BC was assessed *in vitro* and *in vivo* in BALB/c mice via oral and intravenous administration. After 5 h of incubation, TFENs showed cellular uptake of 94.6% and 57.2% in MCF-7 and 4T1 cells, respectively. Interestingly, oral and intravenous administration of TFENs showed similar therapeutic effects, suggesting that the system can survive the harsh environment of the GIT, thereby validating its efficacy. Cell cycle arrest, mitochondrial damage, and oxidative stress were demonstrated in MCF-7 cells and were attributed to ROS produced by TFENs, whereas few ROS were detected in normal tissues. Low Ki67 levels further showed that treatment with TFENs could inhibit tumor relapse and stop BC metastasis (Chen et al., 2022). Thus, the studies above demonstrated that exosomes have great potential to be used for the treatment of both early-stage breast cancer and metastatic breast cancer, in addition to being further translated clinically.

### 4.4 Scaffolds

Scaffolds are injects or implants that are used to deliver drugs, genes, and cells. Natural and synthetic polymers are used to make scaffolds in several forms, including nanofiber matrices, three-dimensional (3D) porous matrices, porous microspheres, and thermosensitive hydrogels (Zielińska et al., 2023). One of the unique characteristics of scaffolds is their porous structure, which allows the diffusion of nutrients, oxygen, and waste products (Yazdanpanah et al., 2022). Scaffolds can be classified based on material composition, biodegradability, implantation mode, and mechanical properties. Hydrogels that undergo a sol-gel transition are often used to design injectable scaffolds, making them suitable for minimally invasive therapies and irregular defects. Implantable scaffolds are composed of bio ceramics and biodegradable polymers, yielding a porous, solid structure with high mechanical stability (Mansouri Moghaddam et al., 2025). Yu et al. (2025) developed an innovative scaffold system that can accommodate molecules of different sizes, including natural antioxidants, chitosan nanofibers, and porous gellan gum sponge. They created a bi-layer green formulation to improve the delivery of curcumin and green tea, using chitosan nanofiber-coated gellan gum sponge. The evaluation of scaffolds’ mechanical features demonstrated their similarity to native breast tissues, with no adverse effects when curcumin and green tea were added. A significant reduction in MCF-7 cell viability was observed, from 92.73% to 23.54%. Each of the encapsulated antioxidants showed different release profiles: curcumin was released more slowly from the nanofibers, while green tea showed rapid release from gellan gum, demonstrating dual release from the scaffold system. Moreover, as a result of encapsulation within a bilayer scaffold, the radical-scavenging properties of the antioxidants were improved, with activity increasing from 62.83% to 95.62%. Overall, the study demonstrated scaffolds’ ability to enhance breast cancer therapy by co-delivering plant-based antioxidants (Yu et al., 2025). Studies on hydrogels, micelles, dendrimers, exosomes, and scaffolds are summarized in Table 4.

**TABLE 4:** Overview of antioxidant loaded drug delivery systems for enhanced breast cancer therapy.

Drug carrier	Material	Antioxidant	Evaluation model	Administration route	Reference
Hydrogel	Hyaluronic acid	Resveratrol	MDA-MB-231, female nude mice	Intra-tumoral	Shin et al. (2022)
Hydrogel	Poloxamer 407	Resveratrol	MCF-7	—	Kotta et al. (2022)
Hydrogel	BSA	Quercetin	MDA-MB-231, MDA-MB 468	—	Kundrapu et al. (2025)
Micelles	Pluronic F127, vitamin E-TPGS	Resveratrol	MCF-7, MDA-MB-231	—	Gregoriou et al. (2021)
Micelles	TPGS, soluplus	Curcumin	MCF-7/adr	oral	Dian et al. (2024)
Dendrimers	PAMAM, graphene oxide	Resveratrol	MCF-7, MDA-MB-231	—	Matiyani et al. (2023)
Exosomes	Milk-derived exosomes	Curcumin, Resveratrol	MCF-7, MDA-MB-231, MCF-10A	—	González-Sarrías et al. (2022)
Exosomes	Tea flower exosomes	Tea plant flower	MCF-7, 4T1, BALB/c mice	Oral, intravenous	Chen et al. (2022)
Scaffold	CS, gellan gum	Curcumin, Green Tea	MCF-7	—	Yu et al. (2025)

## 5 Discussion

Breast cancer has been among the leading causes of mortality among women in recent years due to its complexity, heterogeneity, and drug resistance of tumor cells. Therefore, the treatment of breast cancer is emerging to be more effective and less toxic, requiring the least possible surgical interventions (Shahjahan et al., 2020). Researchers have used various drug delivery systems to reduce chemotherapy side effects and enhance overall patient treatment (Woodring et al., 2023). Drug carriers were not only utilized to encapsulate chemotherapeutic agents but also enhance the delivery of natural antioxidants such as curcumin and thymoquinone which has long shown promise in cancer therapy (Alum et al., 2025; Hashim et al., 2024).

Several studies have shown that plant-based antioxidants are safer than chemotherapy, demonstrating similar or better cytotoxicity in BC cells (Behroozaghdam et al., 2022; Bakhtiari et al., 2023; Zhu et al., 2024). While previous studies demonstrated the ability of exogenous antioxidants to induce apoptosis and prevent metastasis of breast cancer cells, they often exhibit low bioavailability, poor solubility, and lack targeting which shows the importance of using a drug vehicle (Sezgin-Bayindir et al., 2023). As demonstrated previously, drug delivery systems significantly enhanced therapeutic effect of antioxidants against breast cancer by achieving a prolonged release and better tissue distribution. In recent years, nanoparticles have emerged as promising drug vehicles to enhance breast cancer therapy due to their small size and ability to provide sustained release with enhanced cellular uptake. While polymer-based nanoparticles showed high encapsulation efficiency and reduced off-target effects, the release kinetics significantly depend on the polymer used (Herdiana et al., 2023). Metal-based nanoparticles offered more flexibility to control the size and shape of the system as well as enhanced its antioxidant and anticancer effect. However, the use of metal nanoparticles remains challenging due to a higher risk of immunogenicity, cytotoxicity, and environmental accumulation, which can affect human health compared to drug vehicles synthesized from natural polymers (Yetisgin et al., 2020). While a hybrid system is more complex to synthesize, it addresses the instability of metallic nanoparticles. It offers a more robust system combining the properties of both natural polymers and metals (Li et al., 2022b). In contrast, lipid-based nanosystems such as liposomes, micelles, dendrimers, and exosomes have low immunogenicity and high biocompatibility due to their phospholipid content (Shrestha et al., 2014). Exosomes, for instance, are extensions of cell membranes, and the immune system therefore does not treat them as foreign substances, showing no reaction (Skotland et al., 2017). In addition to being less immunogenic, liposomes offer dual loading capacity and are often used in combination therapy to address drug resistance by combining a chemotherapy drug with a natural antioxidant. Interestingly, combining chemotherapy with natural antioxidants has significantly increased treatment efficacy and reduced common chemotherapy side effects. However, it is important to note that unmodified conventional liposomes often have a short half-life and are not stable in the harsh environment of the GIT (Shi Liu et al., 2023; Lee, 2020). Hydrogels, on the other hand, can be synthesized using various stimuli-responsive polymers, including pH- and temperature-sensitive polymers, to protect the drug from enzymes and the acidic pH present in the GIT. The performance of hydrogels, however, is highly dependent on the type of polymer used, its composition, and the administration route (Vegad et al., 2023). While scaffolds offer more tissue-mimicking systems that assist in tissue incorporation, cellular infiltration, and mechanical stability, demonstrating high encapsulation efficiency and biocompatibility, they require surgical intervention (Zielińska et al., 2023).

Only one clinical trial has been conducted to evaluate the efficacy of encapsulated natural antioxidants for breast cancer therapy. Women with breast cancer were given oral nano-curcumin capsules during their chemotherapy using doxorubicin for 6 months. Interestingly, cardiomyopathy, which is common in patients under doxorubicin-based chemotherapy, was not observed in any of the patients taking nano-curcumin supplements (Tohidi et al., 2024). For cancer in general and breast cancer specifically, clinical studies on drug delivery systems loaded with natural antioxidants are very limited. Several key factors affect the clinical translation of drug carriers loaded with plant-based antioxidants including safety concerns, immunological challenges, and tumor microenvironment heterogeneity. Most studies in the literature lack *in vivo* validation, which is essential for toxicity and safety concerns, and the long-term effects of plant-based delivery systems remain unknown, underscoring the need for further research.

All drug delivery systems have both advantages and limitations. Antioxidant-loaded drug delivery vehicles, particularly metal-based and hybrid systems, may cause organ dysfunction and induce oxidative stress in healthy tissues when used long-term, as chronic accumulation can lead to toxicity. Moreover, potential immunogenicity must be carefully examined, as some materials or delivery systems may trigger immune responses; although shown to be safe in short-term studies, multiple doses may be immunogenic. Another factor affecting clinical translation is batch-to-batch variability. Since plant extracts vary by source, processing techniques, and season, and drug vehicles, such as green-synthesized nanoparticles, often show considerable variability in composition, size, and charge. Further optimization is therefore required to refine antioxidant-loaded or derived systems, including their structure and composition, as well as *in vivo* validation. We suggest modifying drug vehicles with ligands and antibodies to enhance selectivity and reduce off-target effects. Overall, more research is required to validate the efficacy and safety of plant-derived antioxidants and antioxidant-loaded systems to support the clinical translation of these strategies for targeted breast cancer therapy.

In the context of oxidative stress, the mechanism by which antioxidants induce ROS-mediated apoptosis in breast cancer cells remains to be further investigated. It is important to note that the literature debates the use of antioxidants alone for cancer treatment. Antioxidants, depending on the biological environment, can act as pro-oxidants, either enhancing tumorigenesis or leading to therapy resistance. N-acetylcysteine (NAC) and vitamin C, for instance, were shown to enhance ROS production in tumor cells at high concentrations, acting as pro-oxidants (Liu et al., 2025). High concentrations of ascorbic acid lead to the formation of oxygen radicals because of catalyzing free transition metal ions, demonstrating a pro-oxidant activity (Kaźmierczak-Barańska et al., 2020). According to the National Institutes of Health, antioxidant studies are ambiguous and fail to provide evidence of a consistent beneficial effect when consumed alone or in pairs. Due to their potential to act as prooxidants under certain conditions and concentrations, the debate over using antioxidants remains (Sotler et al., 2019).

Since antioxidants have demonstrated a dual role, it is important to design drug delivery systems that target a specific ROS level rather than simply increasing ROS levels. The drug vehicles previously reviewed provide targeted, controlled delivery of various antioxidants and can therefore be engineered to control ROS levels at and outside the tumor site. In more detail, the system can be designed to maintain a specific level of ROS at the tumor site, while protecting other organs and tissues from toxic ROS levels. In combination with chemotherapy, antioxidants can enhance treatment efficacy by modulating intratumoral ROS to support pro-apoptotic, chemo-sensitizing effects and reduce off-target toxicity in healthy tissues via controlled delivery. Stoeva et al. (2025) mentioned that green tea, which is recognized as an antioxidant dietary source, exerts pro-oxidant effects depending on multiple factors, including chemical structure, concentration, and cellular microenvironment. The study has further confirmed the relationship between drug dose and antioxidant behavior, in which green tea scavenges ROS at moderate doses and displays pro-oxidant effect at higher doses.

Breast cancer is far more complex and consists of other types, including HER2+ and luminal A/B. However, a significant number of studies demonstrating the effects of antioxidants on breast cancer, including curcumin, thymoquinone, and resveratrol, were evaluated in TNBC (Li et al., 2022a; Bhattacharya et al., 2020; Shin et al., 2022). Thus, we suggest giving greater attention to other BC types by developing antioxidant-loaded drug delivery vehicles that would efficiently work for a specific type or multiple types simultaneously. Drug delivery systems for HER2+ could be improved by using ligands or antibodies that target its receptor, thereby enhancing treatment with natural-product antioxidants. Overall, antioxidant-based drug delivery systems have shown promise in enhancing the treatment of breast cancer and are receiving attention as a natural alternative to chemotherapeutic drugs.
